# Microbial iron metabolism as revealed by gene expression profiles in contrasted Southern Ocean regimes

**DOI:** 10.1111/1462-2920.14621

**Published:** 2019-04-26

**Authors:** Pavla Debeljak, Eve Toulza, Sara Beier, Stephane Blain, Ingrid Obernosterer

**Affiliations:** ^1^ Sorbonne Université CNRS, Laboratoire d'Océanographie Microbienne, LOMIC F‐66650 Banyuls/mer France; ^2^ Department of Limnology and Bio‐Oceanography University of Vienna, A‐1090 Vienna Austria; ^3^ Université Perpignan Via Domitia IHPE UMR 5244, CNRS, IFREMER, Univ. Montpellier, F‐66860 Perpignan France; ^4^ Leibniz Institute for Baltic Sea Research Warnemünde Germany

## Abstract

Iron (Fe) is a limiting nutrient in large regions of the ocean, but the strategies of prokaryotes to cope with this micronutrient are poorly known. Using a gene‐specific approach from metatranscriptomics data, we investigated seven Fe‐related metabolic pathways in microbial communities from high nutrient low chlorophyll and naturally Fe‐fertilized waters in the Southern Ocean. We observed major differences in the contribution of prokaryotic groups at different taxonomic levels to transcripts encoding Fe‐uptake mechanisms, intracellular Fe storage and replacement and Fe‐related pathways in the tricarboxylic acid (TCA) cycle. The composition of the prokaryotic communities contributing to the transcripts of a given Fe‐related pathway was overall independent of the *in situ* Fe supply, indicating that microbial taxa utilize distinct Fe‐related metabolic processes. Only a few prokaryotic groups contributed to the transcripts of more than one Fe‐uptake mechanism, suggesting limited metabolic versatility. Taxa‐specific expression of individual genes varied among prokaryotic groups and was substantially higher for all inspected genes in Fe‐limited as compared to naturally fertilized waters, indicating the link between transcriptional state and Fe regime. Different metabolic strategies regarding low Fe concentrations in the Southern Ocean are discussed for two abundant prokaryotic groups, *Pelagibacteraceae* and *Flavobacteriaceae*.

## Introduction

Since John Martin's ‘iron hypothesis’ was introduced in the late 1980s to solve the high nutrient low chlorophyll (HNLC) paradox in the ocean, the micronutrient iron (Fe) has been recognized as a major factor in the regulation of ocean primary productivity (Martin, 1990; Tagliabue *et al*., 2017). The Southern Ocean as the largest HNLC area has been subject to multiple mesoscale artificial Fe fertilization studies focusing on enhanced phytoplankton blooms through Fe input (reviewed in the study by Boyd *et al*., 2007). Together with investigations in naturally Fe‐fertilized regions (Blain *et al*., 2007; Pollard *et al*., 2009), the control by Fe of primary productivity and subsequent carbon dioxide (CO_2_) drawdown in this ocean has been confirmed.

Heterotrophic microorganisms rapidly respond to phytoplankton blooms induced by Fe‐fertilization (Cochlan, 2001; Hall and Safi, 2001; Oliver *et al*., 2004; Obernosterer *et al*., 2008) and they remineralize a substantial fraction of phytoplankton‐derived dissolved organic matter (Christaki *et al*., 2014). Fe is essential for microbial heterotrophic metabolism, the access to this micronutrient by various taxa will therefore affect the processing of organic carbon. The limited number of measurements indicates that heterotrophic prokaryotes have cellular Fe quotas that are similar or higher than those of phytoplankton (Tortell *et al*., 1999; Sarthou *et al*., 2008; Fourquez *et al*., 2012). The majority of Fe (> 90%) in heterotrophic prokaryotic cells is located in the respiratory chain (Andrews *et al*., 2003) and as a consequence Fe limitation results in a prokaryotic reduction in prokaryotic respiration and growth rates (Tortell *et al*., 1999; Smith *et al*., 2010; Fourquez *et al*., 2014; Koedooder *et al*., 2018). Experimental studies testing the effect of Fe on natural prokaryotic communities have revealed both positive and negative bulk metabolic responses (summarized in the study by Obernosterer *et al*., 2015), likely reflecting temporal and spatial variability of the bioavailability and the cellular requirements of this micronutrient. In the region off Kerguelen Island, heterotrophic prokaryotic growth and production were limited by Fe and organic carbon in early spring (Obernosterer *et al*., 2015), leading to competition between heterotrophic and phototrophic microorganisms for this micronutrient (Fourquez *et al*., 2015).

These observations raise the question of the mechanisms used by microbial taxa to acquire and to metabolize this micronutrient in cellular processes. An increasing number of prokaryotic genomes and of metagenomes originating from global ocean surveys has provided insights to the inventories of Fe‐related pathways (Desai *et al*., 2012; Hopkinson and Barbeau, 2012; Toulza *et al*., 2012; Hogle *et al*., 2016). These studies have shown that the genomic potential for Fe‐uptake mechanisms varies among prokaryotic taxa (Hopkinson and Barbeau, 2012; Hogle *et al*., 2016) and that the prevalence of Fe‐related pathways in prokaryotes reflects Fe concentrations across ocean regions (Toulza *et al*., 2012). The single‐cell approach MICRO‐CARD‐FISH using ^55^Fe revealed that the community taking up Fe in the Southern Ocean was dominated by *Gammaproteobacteria* and FCB, while SAR11 and *Roseobacter* had overall lower contributions (Fourquez *et al*., 2016). Fe limitation has been shown to induce the glyoxylate shunt in heterotrophic bacterial model organisms (Smith *et al*., 2010; Fourquez *et al*., 2014; Koedooder *et al*., 2018), a pattern that was also observed for SAR11 in the HNLC Southern Ocean (Beier *et al*., 2015). The glyoxylate shunt bypasses two decarboxylation steps and the coupled release of CO_2_ and reducing equivalents (NADH_2_) of the TCA cycle, with important consequences on ATP production and processing of organic carbon (Koedooder *et al*., 2018).

The aim of the present study was to extend these observations, by providing a detailed picture on the expression of genes responsible for Fe‐uptake and Fe‐related downstream processes in Southern Ocean natural prokaryotic communities. We screened the total mRNA from metatranscriptomes against a database that contained 10,411 protein sequences corresponding to seven Fe‐related metabolic pathways connected to Fe transport, Fe storage and central carbon metabolism. We explored the functional expression profiles of different taxa in microbial communities originated from HNLC and naturally Fe‐fertilized waters in the region off Kerguelen Island.

## Results

### *Environmental context*

**Figure 1:**
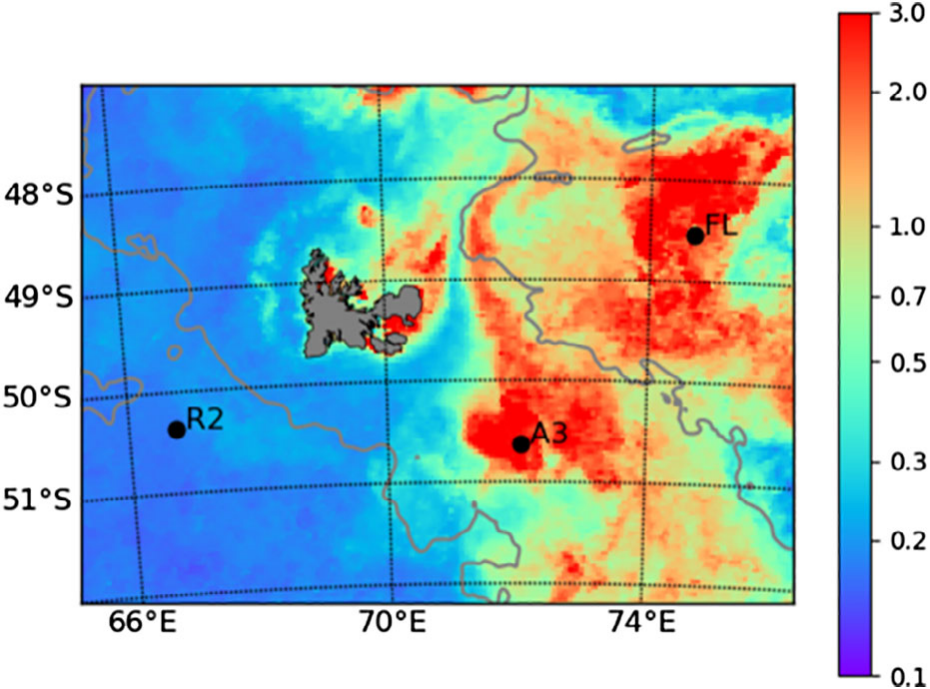
Location of sampling sites positions superimposed on the monthly composite satellite image of chlorophyll (μg L^−1^) provided by Copernicus Marine Service for (November 2011, 4 × 4 km). The grey line denotes 1000 m isobaths.

The three stations considered in the present study were part of the Kerguelen Ocean and Plateau compared study 2 (KEOPS2)‐cruise that took place in November 2011. Station (R‐2) was located in HNLC waters west of Kerguelen Island (Fig. 1), and two stations were situated east of the island in naturally Fe‐fertilized waters south (Station A3‐2) and north (Station F‐L) of the polar front. Concentrations of dissolved Fe in the surface mixed layer varied between 0.13 ± 0.05 nM and 0.22 ± 0.06 nM (Quéroué *et al*., 2015) (Table 1). Chlorophyll *a* concentrations were low at station R‐2 (Chl *a*, 0.25 ± 0.08 μg L^−1^) and up to 16‐fold higher in naturally fertilized waters (Lasbleiz *et al*., 2014) (Table 1). The abundance, production and respiration of heterotrophic prokaryotes were several fold enhanced at the fertilized sites as compared with HNLC waters. Concentrations of DOC, DON and DOP were not enhanced at the Fe‐fertilized sites, probably due to the rapid consumption of organic matter by heterotrophic prokaryotes. Despite these pronounced differences in biomass and production, heterotrophic prokaryotes were shown to be limited by Fe at the HNLC‐site and in Fe‐fertilized waters (Obernosterer *et al*. 2015).

**Table 1:** Location, date, biogeochemical properties and bulk prokaryotic parameters at the three study sites. All parameters are mean ± SD for the surface mixed layer.

	R‐2	F‐L	A3‐2
Latitude S	50.3590	48.5222	51.0333
Longitude E	66.7170	74.6500	72.0833
Date of sampling	26 October 2011	7 November 2011	16 November 2011
Sampling depth (m)	60	20	20
Surface mixed layer (m)	105 ± 15	38 ± 7	153 ± 15
Dissolved and particulate nutrients			
DOC (μM)^a^	47.8 ± 0.4	49.6 ± 1.3	51.3 ± 1.5
DON (μM)^b^	6.1 ± 0.04	5.47 ± 1.33	6.44 ± 2.2
DOP (μM)^b^	0.3 ± 0.02	0.26 ± 0.12	0.36 ± 0.04
POC (μM)^a^	6.5 ± 1.8	11.5 ± 1.2	13.5 ± 1.8
DFe (nmol L^−1^)^c^	0.13 ± 0.05	0.22 ± 0.06	0.16 ± 0.03
Chlorophyll a (μg L^−1^)^d^	0.25 ± 0.08	4.00 ± 1.58	2.03 ± 0.34
Prokaryotic abundance (×10^5^ cells mL^−1^)^e^	2.72 ± 0.3	6.06^c^^,^ f	3.16 ± 0.5
Prokaryotic production (ng C L^−1^ h^−1^)^e^	2.59 ± 0.53	65.7 ± 1.62	19.9 ± 3.4
Prokaryotic respiration (μmol O_2_ L^−1^ d^−1^)^e^	0.25 ± 0.12	1.37 ± 0.64	0.63 ± 0.45

a From Tremblay *et al*. (2015).

b From Blain *et al*. (2015).

c From Queroue *et al*. (2016).

d From Lasbleiz *et al*. (2014).

e From Christaki *et al*. (2014).

f Only one measurement for the surface mixed layer available.

### *Contribution of prokaryotic groups to specific gene expression*

**Figure 2:**
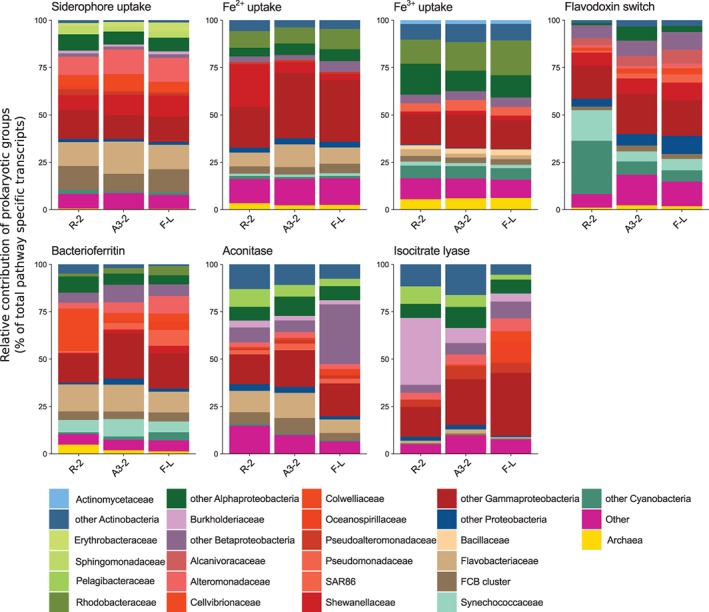
Relative contribution of prokaryotic groups to pathway specific transcripts. Prokaryotic group is defined until taxonomic family level. For clarity, one replicate per station is shown, duplicates as well as mean values and error estimates are shown in the Supporting Information (Fig. S2 and Table S2).

**Figure 3:**
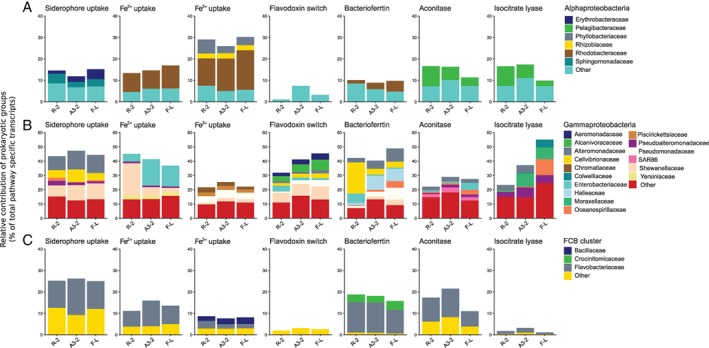
Detailed view of relative contribution of Alpha (A) and Gammaproteobacteria (B) and FCB cluster (C) to pathway specific transcripts. Note the different y‐axis for *Gammaproteobacteria*. For clarity, one replicate per station is shown, duplicates as well as mean values and error estimates are shown in the Supporting Information (Fig. S3 and Table S2).

**Figure 4:**
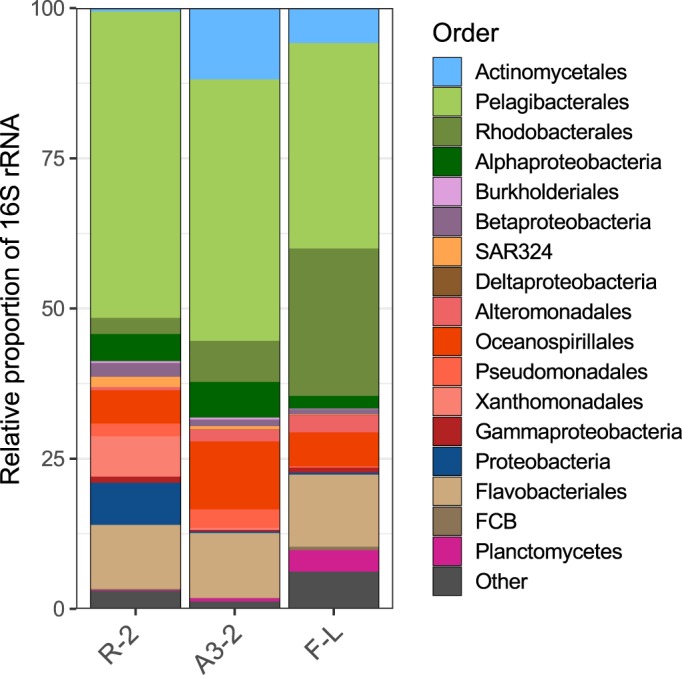
Relative proportion of 16S rRNA gene sequences on the order level (data are from Landa *et al*. 2016).

Filtering and rRNA removal of raw reads resulted in a total of 29–36 Million reads per sample (Supporting Information Table S1). The prokaryotic proportions were calculated for each duplicate based on Blastx results and ranged from 9.7% ± 1.1 at Station R‐2, 14.5% ± 1.0 at A3‐2%, to 19.9% ± 2.9 at F‐L (Supporting Information Fig. S1). The retrieved sequences cover a broad range of families out of 30 bacterial phyla as well as archaea. Highest overall pathway specific contributions were observed for the classes of *Alpha‐, Beta*‐ and *Gammaproteobacteria* as well as the *Fibrobacteres, Chlorobi, Bacteroidetes* (FCB) (Figs. 2 and 3, Supporting Information Figs. S2 and S3, Supporting Information Table S2). These bacterial phyla were shown to be abundant also in data sets derived from 16S rRNA amplicon sequencing (Fig. 4). We observed major differences in the contribution of prokaryotic groups to the different Fe‐uptake mechanisms and this pattern was largely independent of site. Siderophore‐uptake was dominated by *Gammaproteobacteria* (43% of total transcripts) and FCB (25%), while Fe^2+^‐ and Fe^3+^‐uptake revealed an increased contribution of alphaproteobacterial groups, most pronounced for Fe^3+^‐uptake (29%). Within *Gammaproteobacteria* noticeable differences in the contribution of different phylotypes to the three uptake mechanisms could be observed. While *Alteromonadaceae*, *Cellvibrionaceae* and *Shewanellaceae* were abundant contributors to siderophore‐uptake, *Shewanellaceae* and *Enterobacteriaceae*, accounted for most gammaproteobacterial Fe^2+^‐uptake transcripts and *Pseudomonadaceae, Chromatiaceae* and *Piscirickettsiaceae* were major contributors to Fe^3+^‐uptake transcripts (Fig. 3, Supporting Information Table S2). FCB were mostly represented by *Flavobacteriaceae* for siderophore‐ and Fe^2+^‐uptake, and *Bacillaceae* contributed additionally to Fe^3+^‐uptake. Within *Alphaproteobacteria, Rhodobacteracea* substantially contributed to Fe^2+^‐ and Fe^3+^‐uptake, but this group had low siderophore uptake transcripts (<1.6% of alphaproteobacterial transcripts). A contrasting pattern was observed for *Sphingomonadaceae* and *Erythrobacteraceae* that contributed to siderophore uptake transcripts but neither Fe^2+^ nor Fe^3+^. *Pelagibacteraceae* transcripts were not detectable for siderophore‐ and Fe^2+^‐uptake, and Fe^3+^‐uptake transcripts belonging to this group accounted for 0.8% of alphaproteobacterial transcripts. *Actinobacteriaceae* and *Archaea* were almost absent from siderophore‐uptake but contributed to Fe^2+^‐ and Fe^3+^‐uptake.

The flavodoxin switch and bacterioferritin transcripts revealed both high contributions of gammaproteobacterial groups, and smaller proportions of alphaproteobacterial and FCB transcripts, in particular for flavodoxin transcripts. Pronounced differences in the contribution of different gammaproteobacterial phylotypes to the two Fe‐related processes were detectable. *Shewanellaceae, Alcanivoracaceae* and *Aeromonadaceae* were the most important contributors to flavodoxin switch transcripts, while *Cellvibrionaceae, Halieaceae* and *Alteromonadaceae* dominated the bacterioferritin transcripts. *Flavobacteriaceae* and *Rhodobacteraceae* contributed each substantially to bacterioferritin transcripts, while flavodoxin switch transcripts belonging to these groups were almost absent. *Pelagibacterales* did not contribute to flavodoxin switch and bacterioferritin transcripts. *Cyanobacteria* accounted for a large portion of flavodoxin switch transcripts, in particular at the HNLC‐site. The relative contribution of cyanobacteria to 16S rRNA sequences varied between 0.37% at the HNLC and 0.41% at the Fe‐fertilized stations respectively (Landa *et al*. 2016). Taken together, these results construe specific Fe‐uptake and Fe‐processing mechanisms for several prokaryotic groups.

We identified two categories of prokaryotic groups contributing to transcripts of aconitase and isocitrate lyase, genes associated with the TCA cycle. Alphaproteobacterial phylotypes, in particular *Pelagibacteraceae* and *Actinobacteriaceae*, each had similar contribution to aconitase and isocitrate lyase. In contrast, the proportions of transcripts accounted for by *Flavobacteriacaea* and gammaproteobacterial phylotypes varied considerably between the two genes. *Flavobacteriacaea* had higher contributions to aconitase transcripts than to those of isocitrate lyase, and gammaproteobacterial phylotypes, such as *Pseudoalteromonadaceae, Moraxellaceae, Oceanosprillaceae*, *Alteromonadaceae* and *Colwelliaceae* showed the opposite pattern. *Burkholderiaceae*, a group that had low contributions to all other transcripts, accounted for up to 30% of the aconitase and isocitrate lyase transcripts.

### *Transcriptional activity in HNLC and Fe‐fertilized waters*

**Figure 5:**
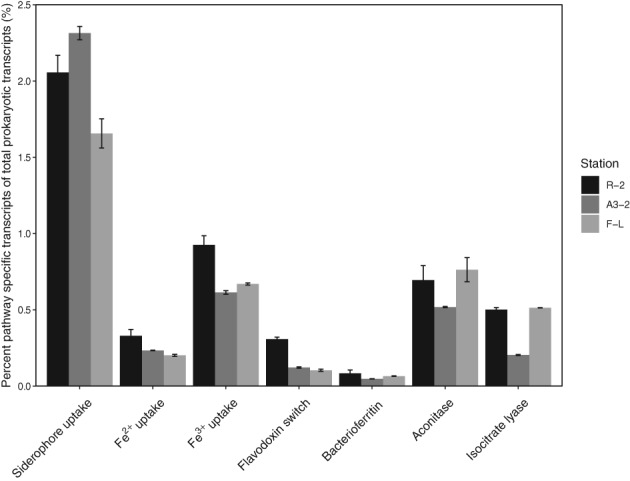
Percent contribution of pathway‐specific transcripts to total prokaryotic transcripts at a given site. Bars represent mean values and error bars represent minimum and maximum values.

Among site comparison of pathway‐specific transcripts revealed that Fe^2+^, Fe^3+^, the flavodoxin switch and bacterioferritin had higher proportions at R‐2 as compared to the Fe‐fertilized sites (Fig. 5). In contrast, no such pattern was observed for siderophore‐uptake, aconitase and isocitrate lyase. Comparison among genes encoding for the different Fe‐uptake mechanisms was possible due to the similar gene length (Supporting Information Table S4). The relative transcript abundance of siderophore‐uptake was roughly 6‐ and 2‐fold higher than those of Fe^2+^‐ and Fe^3+^‐uptake respectively.

**Figure 6:**
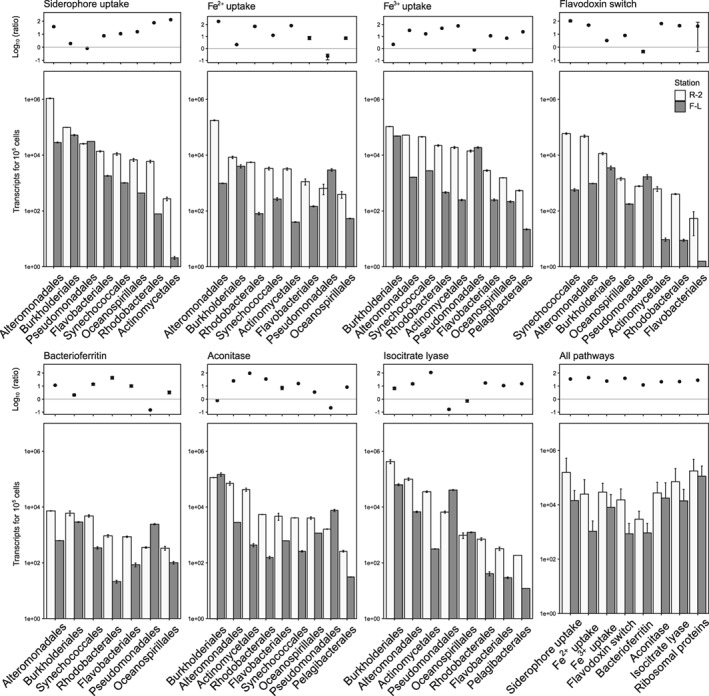
Taxa‐specific transcript abundance (per 10^5^ cells) of a given pathway in HNLC (Station R‐2, white bars) and Fe‐fertilized waters (Station F‐L, grey bars), and the Log_10_ of the ratio between the cell‐specific transcripts at R‐2 to F‐L. The order of prokaryotic groups is from high to low cell‐specific transcripts at Station R‐2. The panel ‘All pathways’ shows the cell‐specific transcripts for all prokaryotic groups combined and the Log_10_ of the R‐2/F‐L ratios for the respective pathways, including ribosomal proteins. Bars represent mean values and error bars represent minimum and maximum values. Error estimates are given in the Supporting Information (Table S3). Ratios were calculated by dividing each duplicate pair.

In order to assess a potential per cell transcriptional activity, results from qPCR expression of isocitrate lyase in *SAR11* at two sites, R‐2 and F‐L, were used as correction factors (see Experimental procedures). This normalization step was chosen in order to answer the following question: Does gene expression vary between HNLC and Fe‐fertilized waters? And further, how variable is cell specific expression of a given gene among the different prokaryotic groups? This step was done for representatives from the prokaryotic phyla (presented in Fig. 2) for which the gene transcripts and 16S relative abundances were available for a given phylogenetic level (Fig. 4 and Supporting Information Fig. S5). To verify the approach, ribosomal proteins, essential in cellular processes of translation, were retrieved from Kyoto Encyclopedia of Genes and Genomes (KEGG) and screened against our data sets (Fig. 6 and Supporting Information Fig. S4).

Cell‐specific expression of all genes considered in the present study was considerably higher (10‐ to 1000‐fold) at Station R‐2 in comparison to F‐L for all prokaryotic groups (Fig. 6 and Supporting Information Table S3). Exceptions were *Pseudomonadales* that consistently revealed an inverse pattern, and *Burkholderiales* for which differences between sites were in most cases small. In comparison, the ribosomal proteins showed the lowest fold‐change (1.5) between R‐2 and F‐L. For clarity, the following comparison of taxa‐specific expression levels among prokaryotic groups is focused on the HNLC site R‐2. *Alteromonadales* had the highest taxa‐specific expression levels for all genes, while taxa‐specific expression levels of a given gene was more variable for the other prokaryotic groups. *Flavobacteriales*, *Oceanospirillales* and *Rhodobacterales* had similar taxa‐specific siderophore‐uptake expression while *Rhodobacterales* and *Actinomycetales* had substantially higher taxa‐specific Fe^3+^‐uptake as compared with *Flavobacteriales* (8‐ to 6‐fold respectively) and *Oceanospirillale*s (15‐ to 12‐fold)*. Rhodobacterales, Flavobacteriales* and *Oceanospirillale*s had similar bacterioferritin expression levels, and flavodoxin switch expression levels belonging to *Flavobacteriales* were considerably lower than those of the other groups. Taxa‐specific gene expression of aconitase was similar for *Rhodobacterales, Flavobacteriales* and *Oceanospirillale*s. In contrast, taxa‐specific expression levels of isocitrate lyase were highly variable among groups, with *Actinomycetales* and *Pelagibacterales* at the higher and lower ranges respectively.

## Discussion

We present here taxon‐specific strategies of Fe‐uptake and intracellular processes dependent on Fe, a crucial, yet growth‐limiting element for microbial heterotrophs in large areas of the ocean. By investigating the *in situ* expression patterns of candidate genes from metatranscriptomics data, we provide novel insights into these metabolic traits of Southern Ocean microbial communities in contrasting Fe‐ and C‐regimes. We observe major differences in the contribution of prokaryotic groups to the pathways investigated, indicating distinct metabolic capabilities for Fe‐related processes and downstream carbon metabolism for microbial taxa. Our finding that taxa‐specific expression levels are substantially higher under Fe‐limited conditions suggests that the transcriptional states related to Fe‐uptake and Fe cell‐content reduction or control are associated with the *in situ* Fe‐supply.

Our observation that *gamma*‐ and *flavobacterial* groups accounted for a large fraction of siderophore‐uptake transcripts, while *alphaproteobacterial* groups had an increased contribution to Fe^3+^‐uptake transcripts corroborates previous findings on the genomic potential of representatives of these classes (Hopkinson and Barbeau, 2012; Tang *et al*., 2012; Hogle *et al*., 2016). A survey of 206 bacterial genomes revealed that Ton‐B‐dependent transporters (TBDTs), many of which are known as siderophore type transporters, are common in *Gammaproteobacteria* and *Bacteroidetes* but less abundant in *Alphaproteobacteria*, and absent in *Pelagibacter ubique* (Hopkinson and Barbeau, 2012; Tang *et al*., 2012). Within *Alphaproteobacteria*, *Erythrobacteracea* and *Sphingomonadacea* were major contributors to siderophore‐uptake transcripts in the present study, an observation supported by the genomic potential of representative strains (Tang *et al*., 2012). Fe^3+^‐uptake gene expression revealed a contrasting pattern, with high contributions of *Rhodobacteraceae* and *Actinomycetales* and a minor contribution of FCB. While Fe^3+^ transporters were abundant in many of the 206 bacterial genomes investigated, only 1 of 16 *Bacteroidetes* genomes contained this type of transporter (Hopkinson and Barbeau, 2012). Despite its dependency on inorganic Fe‐uptake, *Pelagibacteraceae* had a minor contribution to the overall Fe^3+^‐uptake gene expression (0.8% of total Fe^3+^ transcripts) and the lowest cell‐specific Fe^3+^‐uptake (Fig. 6). The lower copy numbers of Fe^3+^ transporters in *SAR11* as compared with *Roseobacter* genomes (Hogle *et al*., 2016) and potentially lower Fe requirements could explain this observation.

The dominating microbial contributors to the transcripts of a given Fe‐uptake mechanism were overall similar among sites and thus largely independent of the *in situ* Fe supply. This contrasts with the different relative contributions of the observed groups at the sites (Fig. 4, Landa *et al*., 2016) and suggests that our observations on the transcriptome level are mainly driven by the metabolic potential of the respective prokaryotic groups (see Supporting Information Fig. S5). In support of this conclusion, our results illustrate that only a few prokaryotic groups contribute substantially to the transcripts of more than one Fe‐uptake mechanism. This was the case for *Shewanellaceae* transcripts that were present for the three Fe‐uptake mechanisms, and for *Rhodobacteraceae* and the FCB cluster that accounted both for substantial proportions of Fe^2+^‐ and Fe^3+^‐uptake transcripts.

The 2‐ to 6‐fold higher proportions of siderophore‐uptake gene expression as compared with Fe^3+^ and Fe^2+^ could indicate that this mechanism was a more efficient pathway for prokaryotes to acquire Fe. Most dissolved Fe (99%) (Gledhill and van den Berg, 1994; Rue and Bruland, 1995) is complexed by organic ligands leaving extremely low steady‐state concentrations of inorganic Fe^3+^ noted as Fe’. The steady‐state concentrations of Fe^2+^ are also typically extremely low in oxygenated surface waters although reduction mediated by different processes could locally produce enhanced concentrations. Fe’ has been considered a more bioavailable and thus more important form than siderophore‐bound Fe for phytoplankton (Morel *et al*., 2008; Lis *et al*., 2015). Our observations suggest that heterotrophic prokaryotes favour the uptake of Fe bound to siderophores, which presents several advantages. First, once complexed by siderophores, Fe is hardly available for most phytoplankton (Lis *et al*., 2015), even though the idea that phytoplankton cannot directly use Fe‐siderophore complexes has recently been challenged (Kazamia *et al*., 2018; McQuaid *et al*., 2018). Heterotrophic prokaryotes thereby avoid competition with phytoplankton for this scare resource. Second, siderophores can contribute to access initially not available forms of Fe, such as particulate Fe or Fe‐organic complexes (Kraemer, 2004). Third, Fe‐siderophore‐uptake could be stimulated by siderophore‐production of the same microbial cell and thus provide an advantage to certain taxa (Martinez *et al*., 2003; Hopkinson and Barbeau, 2012; Sijerčić and Price, 2015; Boiteau *et al*., 2016). Our taxa‐specific transcripts point out that the expression of the genes encoding for siderophore‐uptake was increased (20‐ to 135‐fold) under Fe‐limited conditions when the competition for the acquisition of this resource was highest. This latter observation agrees well with the previously reported global scale inverse relationship between siderophore‐uptake gene occurrence and Fe concentrations (Toulza *et al*., 2012). Under strong Fe limitation, the Fe‐uptake rate can only be increased by the number of Fe transporters as has been demonstrated for phytoplankton (Hudson and Morel, 1993). Our observations point to a similar conclusion for heterotrophic prokaryotes. Besides the capacity to optimize Fe acquisition in limited conditions, the second global strategy for prokaryotes is to decrease the Fe cellular content.

To explore differences in intracellular Fe‐regulatory mechanisms among prokaryotic groups, we investigated the two genes encoding for flavodoxin and bacterioferritin. The non‐Fe containing protein flavodoxin is an iso‐functional protein, which can replace ferrodoxin, an electron shuttle harbouring Fe‐sulfur clusters. The expression of this protein by marine autotrophic plankton was proposed as a proxy for Fe scarcity in the oceans (Roche *et al*., 1996), and insight into the mechanisms of how Fe availability regulates this protein was obtained from studies on various temporal and spatial scales (Erdner *et al*., 1999; Saito *et al*., 2011; Tara Oceans Coordinators *et al*., 2018). A metagenomic analysis revealed that prokaryotes lacking this flavoprotein are confined to coastal areas where Fe supply is high, while flavodoxin‐containing marine prokaryotes are preferably located in open ocean sites (Toulza *et al*., 2012). Given the roughly 35‐fold higher proportion of total flavodoxin transcripts and the higher cell‐specific expression in HNLC waters as compared with Fe‐fertilized sites, our results extend the understanding of the regulation of this switch by Fe‐availability for a large range of prokaryotic groups. In the present study, the dominant contributors were *gammaproteobacterial* taxa and Cyanobacteria, while *Alphaproteobacteria* and the FCB cluster had minor contributions to flavodoxin transcripts. These latter groups revealed also lowest cell‐specific expression patterns, suggesting that many FCB and *alphaproteobacterial* members might make use of other strategies to cope with Fe‐limitation.

Ferritins are compounds that were shown to regulate the storage and the release of intracellular Fe in a number of eukaryotic microorganisms (Marchetti *et al*., 2009; Botebol *et al*., 2015). Bacterioferritins are known to be involved in the storage of Fe in *Bacteria*; however, the regulation and the exact physiological mechanisms of these compounds are not clear (Andrews *et al*., 2003; Carrondo, 2003). In the GOS data set, bacterioferritin gene abundance was higher at coastal sites with overall high Fe concentrations (Toulza *et al*., 2012). In the present study, all prokaryotic groups contributed to bacterioferritin transcripts. The differences in bacterioferritin transcripts between HNLC and Fe‐fertilized waters were far less pronounced than the other Fe‐related metabolisms. This could indicate that bacterioferritin‐related processes are occurring at background levels, for instance, as a control of Fe homeostasis, rather than as storage of Fe in response to episodic Fe supply.

The higher cell‐specific expression of siderophore‐, Fe^2+^‐, Fe^3+^‐uptake, flavodoxin and bacterioferritin transcripts in HNLC as compared with Fe‐fertilized waters highlight the increased investment in Fe‐related metabolism when prokaryotic growth and production are limited by Fe and organic carbon (Obernosterer *et al*., 2015). But how can this affect cellular carbon metabolism? We addressed this question by investigating the two enzymes aconitase and isocitrate lyase belonging to a central metabolic pathway, the TCA cycle. Aconitase, an Fe‐containing enzyme, transforms citrate to isocitrate, which can either serve as a substrate for the enzyme isocitratedehydrogenase (IDH) in the TCA cycle or as a substrate for isocitrate lyase, a non‐Fe‐containing enzyme that induces the glyoxylate shunt, a bypass of the TCA cycle (Supporting Information Fig. S6). While the regulation of the glyoxylate shunt can be driven by a number of factors, its induction by Fe‐limitation has been demonstrated in bacterial model organisms (Fourquez *et al*., 2014; Koedooder *et al*., 2018). Using genetic tools and bioreporters demonstrated that the isocitrate lyase knock‐out strains of gammaproteobacterium *Photobacterium angustum* S14 had significantly lower growth and respiration rates as compared with the wild type under Fe‐limited conditions (Koedooder *et al*., 2018). Using qPCR, *SAR11* cell‐specific isocitrate lyase gene expression was higher at Station R‐2 as compared with F‐L (Beier *et al*., 2015). The increased cell‐specific isocitrate‐lyase expression in Fe‐limited as compared with Fe‐fertilized waters observed in the present study extends this previous observation to several prokaryotic groups.

In the context of these recent findings, we focus in the following discussion on two prokaryotic groups with distinct patterns in the expression of aconitase and isocitrate lyase. *Pelagibacteraceae* contributed similarly to the expression of both genes; in contrast, *Flavobacteriaceae* revealed substantially higher contributions to aconitase (7%–13% of prokaryotic transcripts) as compared with isocitrate lyase transcripts (0.7%–2.2% of prokaryotic transcripts). In addition, *Flavobacteriaceae* had 18.5‐fold higher cell‐specific aconitase expression than *Pelagibacteraceae*, but both groups had similar cell‐specific isocitrate lyase expression. These observations could indicate that the entire TCA cycle is more preferentially used in *Flavobacteriaceae* than in the members of *Pelagibacteraceae* with consequences on the production of NADH and ATP equivalents.

The combined information obtained by the present results and previous knowledge on characteristics of these bacterial groups leads us to propose two distinct ecological strategies with respect to Fe‐related processes for *Pelagibacteraceae* and *Flavobacteriaceae*. Members of *Pelagibacteraceae* appear to be the thriftiest group, characterized by the unique use of Fe^3+^‐uptake, performed by ABC‐type transporters that do not require the costly outer membrane receptors (Andrews *et al*., 2003) but do require ATP. Additionally, members of this group lack Fe storage and the flavodoxin switch for which no transcripts were detectable in the present study. Despite its dependency on Fe^3+^‐uptake, *Pelagibacteraceae* had a minor contribution to the total transcripts, suggesting low Fe requirements of this group. These characteristics extend those described previously of the most prominent representatives such as the streamlined *SAR11* (reviewed in the study by Giovannoni, 2017). The *SAR11* clade has been shown to possess high‐affinity uptake systems for a range of small molecules present at low concentrations, including two‐carbon compounds, known to induce the glyoxylate shunt. This strategy allows to maintain cellular metabolism with low Fe requirements and to efficiently metabolize small molecules.

Members of *Flavobacteriaceae* appear to be characterized by different features. Our results point out that members of this group display the most competitive Fe‐uptake systems. Also, they can potentially regulate homeostasis with bacterioferritin in particular under Fe limited conditions. This group has a moderate use of the glyoxylate shunt, because the biosynthesis of TBDTs, and their transport to the cytoplasm renders the acquisition of siderophore‐bound Fe a process that is costlier in terms of energy and carbon requirements than that of Fe^3+^‐uptake. Siderophore biosynthesis, coupled in many bacterial genomes to TBDTs (Hopkinson and Barbeau, 2012), adds further energy requirements (Sijerčić and Price, 2015). Besides Fe‐siderophore‐uptake, TBDTs were associated with the uptake of a range of substrates, such as carbohydrates, amino acids, amino sugars or vitamin B_12_ (Schauer *et al*., 2008; Noinaj *et al*., 2010). The highest number and most diverse types of TBDTs were associated with *Gammaproteobacteria* and *FCB* in the GOS data set (Tang *et al*., 2012). This genomic information, in combination with whole genome sequencing, culture‐based studies and single‐cell approaches have led to the characterization of members of FCB to be efficient degraders of polymeric organic matter (Kirchman *et al*., 2003; Bauer and Blodau, 2006; Kabisch *et al*., 2014). Even though members of both groups are well equipped to thrive in Fe‐limited environments, *Pelagibacteraceae* are likely to have an advantage over *Flavobacteriaceae* when organic carbon is limiting. These contrasting characteristics for members of *Pelagibacteracea* and *Flavobacteriaceae* extend those known for other metabolic traits to clear prevalence for Fe^3+^‐ and siderophore‐uptake, respectively, and could be considered as ecological strategies in an ocean region where microbial activity is limited by Fe and organic carbon.

## Experimental procedures

### *Sample collection*

Seawater samples were collected during the KEOPS2 cruise (Kerguelen Ocean and Plateau Compared Study 2, 8 October to 30 November2011) in the Indian sector of the Southern Ocean.

Seawater samples were collected with 12 l Niskin bottles mounted on a rosette equipped with a CTDO Seabird SBE911‐plus. For nucleic acid extractions, seawater was sampled at one depth in the surface mixed layer, and the chemical and biological parameters were collected throughout the water column (Christaki *et al*., 2014; Lasbleiz *et al*., 2014; Blain *et al*., 2015; Quéroué *et al*., 2015; Tremblay *et al*., 2015) (Table 1).

### *RNA extraction*

For RNA extractions, volumes varying between 15 l and 30 l of pre‐filtered water (200 μm nylon screen and 5 μm polycarbonate isopore filters) were collected onto 0.2 μm SuporPlus Membranes using a 142 mm filtration system (geotech equipment) and a peristaltic pump. The filtration procedure did not exceed 10 min and 10 ml of RNA‐later was added before storage at −80°C. All nucleic acid extractions were performed in triplicates by cutting the filter in three parts.

Total prokaryotic and eukaryotic RNA was extracted using the NucleoSpin® RNA Midi kit (Macherey‐Nagel, Düren, Germany). Filters stored in RNA later were defrosted, removed from the RNA later solution, refrozen in liquid nitrogen and shattered using a mortar. The obtained ‘powder‐like’ filter‐pieces were added together with low binding zirconium beads (OPS Diagnostics, Lebanon, NJ, USA) to the denaturing lysis buffer supplied by the NucleoSpin® RNA Midi kit and cells were disrupted by vortexing for 2 min. Beads were discarded by centrifugation. The extraction with the NucleoSpin® RNA Midi kit include an on‐column DNA digestion step. However, in order to ensure the absence of DNA in the sample, a control PCR reaction was performed without the retrotranscription (RT) step. Samples with DNA contamination, as indicated by amplification products were treated with a second DNA digestion step using the Turbo DNA‐*free* kit (Ambion Life Technologies, Carlsbad, CA, USA). This additional DNAse treatment was followed by purification with the RNeasy MinElute Clean Up kit (Qiagen, Hilden, Germany). The extracted RNA was quantified with the Agilent 2100 Bioanalyzer/Agilent RNA 6000 Nano Kit (Agilent, Santa Clara, CA, USA) and duplicates were chosen for sequencing.

### *Library preparation and sequencing*

Prior to sequencing, ribosomal RNA was treated enzymatically with the RiboZero rRNA stranded RNA protocol to ensure sequencing of primarily messenger RNA followed by cDNA library construction using Illumina TruSeq Stranded mRNA Library Prep kit (Fasteris SA). Libraries were sequenced using paired‐end 2 × 125 read length on one Illumina HiSeq 2500 lane.

### *Bioinformatic analysis*

The raw Illumina reads were checked with FastQC (Andrews 2010; http://www.bioinformatics.babraham.ac.uk/projects/fastqc) and adapters were eliminated using Cutadapt (Martin, 2011). Remaining ribosomal RNA sequences were removed by the riboPicker (Schmieder *et al*., 2012) tool and sequences were checked by interlacing and de‐interlacing paired‐end reads ensuring that the same sequences were removed from each R1 and R2 files and finally retaining only R1 (performed in Galaxy, Afgan *et al*., 2016). Randomized subsets of 1% of the data were affiliated using BLASTX (Altschul *et al*., 1990) against the non‐redundant (nr) protein database followed by the visualization in MEGAN6 (Huson *et al*., 2007) and proportions of unassigned to not assigned sequences as well as prokaryotic to eukaryotic sequences were retrieved (Supporting Information Fig. S1). These sequence data have been submitted to the EMBL databases under accession number PRJEB30315.

### *Database construction*

A database containing sequences of genes involved into Fe‐related metabolic pathway was retrieved from the study by Toulza *et al*. (2012). This database was constructed by screening for bacterial sequences from NCBI with the gene name as query, as well as the protein sequences from the Moore Microbial Genome database (http://www.moore.org/microgenome/) for genes involved in Fe metabolism. For the purpose of this study, the specific sequences for the following pathways were retrieved from the database and updated by searching for these in NCBI protein clusters: Flavodoxin switch (FL), Fe^2+^‐uptake (F2), Fe^3+^‐uptake (F3), siderophore‐uptake (SU), and storage (ST) (Supporting Information Table S2). These five together are further named as ‘Fe’ database.

In addition, protein sequences for two supplementary enzymes were chosen for pathway specific analysis. Aconitase that catalyses the isomerization of citrate to isocitrate via cis‐aconitate in the tricarboxylic acid cycle and isocitrate lyase an enzyme in the glyoxylate shunt which catalyses the cleavage of isocitrate to succinate and glyoxylate. For maximal phylogenetic coverage, all available bacterial protein sequences were retrieved from protein clusters (proteins grouped on taxonomic groups which are non‐redundant) using the NCBI search tool with the protein name as query.

In order to include more sequences from environmental marine bacteria the two databases were aligned using BLASTX tool (Altschul *et al*., 1990) against the Global Ocean Sampling (GOS) protein database (retrieved from /iplant/home/shared/imicrobe/projects/26/CAM_PROJ_GOS.read_pep.fa, Yooseph *et al*., 2007) containing peptide sequences predicted from long reads from Sanger sequencing and thus more robust for the purpose of the analysis. The retrieved GOS sequences were then checked against the KEGG (Kanehisa and Goto, 2000; Kanehisa *et al*., 2016; 2017) and for each custom database sequences with an e‐value of <1e^−5^ were chosen for further analysis and annotated from their KEGG‐Id (Supporting Information Table S3). Finally, each database contained FASTA sequences with the taxonomic affiliation in the header as well as the KEGG‐Id. For SU only Fe‐related siderophore KEGG‐Ids were retained.

Additionally, all ribosomal protein sequences (*n* = 261 980) stored in the KEGG database (download January 2018) were broadly (order level) annotated by retrieving taxonomic information for sequences from KEGG. These sequences were used to recruit ribosomal protein transcripts from our metatranscriptome data that served as verification of the normalization approach.

### *Sequence alignment*

The final curated databases (*n* = 4, Fe, aconitase, isocitrate lyase and ribosomal proteins) containing information on the annotated taxonomic levels (phylum, class, order, family and genus) were aligned to the short‐read translated DNA query sequences for each station and duplicate using diamond blastx (parameters used ‐k 1 ‐e 10 ‐p 12) (Buchfink *et al*., 2015). Total counts per phylum, class, order, family and genus for each database, and in case of Fe, for each pathway, were summed and relative proportions to all prokaryotic reads for bacterial groups were calculated (Supporting Information Fig. S7). Bacterial groups defined at the taxonomic order level with the highest abundances of pathway specific transcripts were taken for further analysis.

### *Normalization approach*

In an attempt to estimate the absolute number of transcripts per sample, we followed the principals published elsewhere (Satinsky *et al*., 2013): the number of reads per sample is normalized by the number of reads obtained from an internal standard added with a known number of RNA molecules to the RNA extraction. However, in our case, we did not add internal standard RNA molecules, but instead based our calculations on the number of SAR11 isocitrate lyase transcripts for normalization, which were quantified earlier via qPCR (Beier *et al*., 2015).

While the number of SAR11 isocitrate lyase transcripts per L water derived from the qPCR approach might be biased, for instance, due to primer miss matches, such biases are strongly reduced for the ratio of SAR11‐isocitrate lyase gene transcripts to SAR11‐isocitrate lyase gene copies (Beier *et al*., 2015). For the normalization step, we therefore assumed that the above‐mentioned ratio derived from qPCR data equals the ratio of SAR11‐isocitrate lyase gene transcripts (metatranscriptome; RNA) to the number of SAR11 cell per L estimated by CARD‐FISH (as described in the study by Fourquez *et al*. 2016):

(1) $${\mathrm{qT}}_{\mathrm{iso}}/{\mathrm{qC}}_{\mathrm{iso}}={\mathrm{mT}}_{\mathrm{iso}}/{\mathrm{nC}}_{\mathrm{SAR}11}$$

where, for the purpose of this study, qT_iso_/qC_iso_ is the ratio of SAR11‐isocitrate lyase gene transcripts L^−1^ (qT_iso_) to SAR11‐isocitrate lyase gene copies L^−1^ (qC_iso_) estimated by qPCR (Beier *et al*., 2015), mT_iso_ is the number of SAR11‐isocitrate lyase gene transcripts L^−1^, and nC_SAR11_ is the SAR11 cells L^−1^ estimated by CARD‐FISH (Fourquez *et al*. 2016)

The ratio qT_iso_/qC_iso_ as well as nC are known variables and Eq. 1 can accordingly be resolved by mT_iso_. We subsequently related mT_iso_ to the number of metatranscriptome reads coding for SAR11 isocitrate lyase gene transcripts (mT_iso_) and used this factor to estimate the absolute transcript numbers per L water or all remaining genes (mT).

qPCR data were only available for Station R‐2 and F‐L, thus samples from A3‐2 were excluded from these calculations. Operational taxonomic units (OTUs) obtained by 16S rRNA gene sequencing were retrieved for Station R‐2 and F‐L from an already published data set from the same sampling date (Landa *et al*., 2016). The OTUs were corrected for copy numbers of 16S rRNA gene per cell per specific taxa obtained from the ribosomal RNA database (Stoddard *et al*., 2015). Total cell numbers per bacterial group were then calculated with 16S rRNA gene relative proportions and counts from the Eub228‐I, ‐II and ‐II catalysed reporter deposition–fluorescence *in situ* hybridization (CARD‐FISH) probe from the same samples (data from the study by Fourquez *et al*., 2016, Supporting Information Table S6).

In order to obtain the number of transcripts per cell, we divided mT for the inspected genes derived from a certain bacterial group (based on the number of hits from the specific databases) by the total cell numbers per L for the respective bacterial group shown in Supporting Information Table S6. While we tried to minimize biases introduced by the qPCR approach, it should be considered that our method is not fully free of biases, also because additional biases might be introduced by combing multiple techniques, such as qPCR, CARD‐FISH and amplicon sequencing data. Accordingly, the retrieved and presented values (Fig. 6) represent a rough estimate of transcripts per cell.

Analysis and visualization of data were performed in the R language [https://cran.r-project.org/, version 3.4.0 (21 April 2017)] using customized colour palettes. Following packages and versions were used: phyloseq_1.22.3, gdtools_0.1.7, gridExtra_2.3, cowplot_0.9.3, gtable_0.2.0, ggpubr_0.2, magrittr_1.5, ggplot2_3.0.0, plyr_1.8.4. Figures were using the open‐source vector graphics editor Inkscape (http://inkscape.org/). Codes and databases are provided through the following link https://github.com/PavlaDe/emi14621/.

## Supporting information


**Appendix S1.** Manuscript_Codes

**Supplementary Figure 1.** Blast results of subsets for assigned to non‐assigned reads.

**Supplementary Figure 2.** Relative contribution of prokaryotic groups to pathway specific transcripts. Prokaryotic group is defined until taxonomic family level. Number 1 and 2 refer to the sequencing results of duplicates per station.

**Supplementary Figure 3 A‐C.** Detailed view of relative contribution of *Alpha‐ and Gammaproteobacteria* and FCB cluster to pathway specific transcripts. Note the different y‐axis for *Gammaproteobacteria*. Number 1 and 2 refer to the sequencing results of duplicates per station.

**Supplementary Figure 4.** Percentage of ribosomal protein transcripts to all prokaryotic transcripts.

**Supplementary Figure 5 A‐G.** Relative contribution of prokaryotic groups to pathway specific transcripts and relative abundance of 16S rRNA gene sequences. Prokaryotic group is defined until taxonomic order level.

**Supplementary Figure 6.** Simple illustration of the glyoxylate shunt (inside of the circle) in which isocitrate lyase cleaves isocitrate into glyoxylate and succinate.

**Supplementary Figure 7.** Detailed plots for duplicates on the phylum, class and order level for each pathway.

**Supplementary Table 1.** General information on sequencing results and reads.

**Supplementary Table 2.** Relative contribution of prokaryotic groups to pathway specific transcripts. For each group, mean value ± standard deviation of 2 replicates are shown. Error estimates are provided for all prokaryotic groups illustrated in Figs 2 & 3, and groups are listed by alphabetic order.

**Supplementary Table 3.** Taxa‐specific transcript abundance (per 10^5^ cells) of a given pathway at Station F‐L and R‐2. For each group, mean value ± standard deviation of 2 replicates are shown. Error estimates are provided for all prokaryotic groups illustrated in Figs 6, and groups are listed by alphabetic order.

**Supplementary Table 4.** Information of databases constructed or modified from Toulza *et al*. (2012) and retrieved by NCBI

**Supplementary Table 5.** Pathways and corresponding KEGG‐Id numbers that were chosen for further analysis

**Supplementary Table 6.** Cells per L for prokaryotic groups in Fig. 6 for station R‐2 and F‐L, calculated as described in experimental procedures.
