# Supplementary material for: Microbial iron metabolism as revealed by gene expression profiles in contrasted Southern Ocean regimes
Source: Environ Microbiol. 2019 Apr 26;21(7):2360–74. doi: 10.1111/1462-2920.14621 (PMC6618146; doi:10.1111/1462-2920.14621)
Supplement: Supplementary file 9 — Supplementary Table 1. General information on sequencing results and reads. [file EMI-21-2360-s009.docx]

| Sample Names | Raw Reads from paired-end sequencing (fastq replicates) | Interlaced reads after trimming and rRNA removal | Percent of prokaryotic reads from BLAST (%) | Number of prokaryotic reads from total reads |
| --- | --- | --- | --- | --- |
| R_1 | 25 795 611  25 795 611 | 31 872 360 | 8.9 | 2 860 032 |
| R_2 | 24 750 021  24 750 021 | 31 519 356 | 10.5 | 3 308 037 |
| A3_2_1* | 23 871 679  23 871 679 | 29 824 202 | 15.3 | 5 514 089 |
| A3_2_2* | 21 853 684  21 853 684 | 34 620 685 | 13.8 | 4 438 123 |
| FL_1* | 20 460 960  20 460 960 | 36 070 585 | 18.0 | 5 373 036 |
| FL_2* | 23 556 644  23 556 644 | 32 099 894 | 21.7 | 7 504 148 |

* The higher number of prokaryotic reads and their higher relative contributions to total reads at A3-2 and F-L as compared to R-2 can be explained by the elevated prokaryotic cell abundances at these sites (Table 1) and differences in the phytoplankton community composition. While larger diatom cells, abundant at the 2 bloom sites, were retained by the 5 µm filter used for the pre-filtration step, phytoplankton biomass at station R-2 was dominated by picoeukaryotes (Lasbleiz et al. 2016) that pass this pore size resulting in a higher number of eukaryote-assigned reads.
