# Supplementary material for: Microbial iron metabolism as revealed by gene expression profiles in contrasted Southern Ocean regimes
Source: Environ Microbiol. 2019 Apr 26;21(7):2360–74. doi: 10.1111/1462-2920.14621 (PMC6618146; doi:10.1111/1462-2920.14621)
Supplement: Supplementary file 10 — Supplementary Table 2. Relative contribution of prokaryotic groups to pathway specific transcripts. For each group, mean value ± standard deviation of 2 replicates are shown. Error estimates are provided for all prokaryotic groups illustrated in Figs 2 & 3, and groups are listed by alphabetic order. [file EMI-21-2360-s010.docx]

**Supplementary Table 2.** Relative contribution of prokaryotic groups to pathway specific transcripts. For each group, mean value ± standard deviation of 2 replicates are shown. Error estimates are provided for all prokaryotic groups illustrated in Figures 2 & 3, and groups are listed by alphabetic order.

| Station | Prokaryotic group | Aconitase | F^2+^ uptake | F^3+^ uptake | Flavodoxin switch | Bacterioferritin | Isocitrate lyase | Siderophore uptake |
| --- | --- | --- | --- | --- | --- | --- | --- | --- |
| A3_2 | Actinomycetaceae | 0 | 0 | 1.9 ± 0.09 | 0 | 0 | 0 | 0 |
| A3_2 | Aeromonadaceae | 0 | 0 | 0 | 3.33 ± 0.16 | 0 | 0 | 0 |
| A3_2 | Alcanivoracaceae | 0 | 0 | 0 | 5.18 ± 0.07 | 0 | 0 | 0 |
| A3_2 | Alteromonadaceae | 3.11 ± 0.06 | 0 | 0 | 1.48 ± 0.21 | 5.04 ± 0.82 | 5.45 ± 0.26 | 13.17 ± 0.25 |
| A3_2 | Aphanizomenonaceae | 0 | 0 | 0 | 4.37 ± 0.24 | 0 | 0 | 0 |
| A3_2 | Archaea | 0 | 2.42 ± 0.13 | 6.15 ± 0.19 | 2.33 ± 0.04 | 1.74 ± 0.21 | 0 | 0.26 ± 0.01 |
| A3_2 | Bacillaceae | 0 | 0 | 2.63 ± 0.11 | 0 | 0 | 0 | 0 |
| A3_2 | Burkholderiaceae | 2.49 ± 0.14 | 0 | 0 | 0 | 0 | 7.45 ± 0.65 | 1.12 ± 0.01 |
| A3_2 | Cellvibrionaceae | 0 | 0 | 0 | 2.55 ± 0.12 | 4.08 ± 0.05 | 0 | 8.81 ± 0.14 |
| A3_2 | Chromatiaceae | 0 | 0 | 2.92 ± 0.34 | 0 | 0 | 0 | 0 |
| A3_2 | Chromobacteriaceae | 0 | 0 | 0 | 0 | 1.72 ± 0.48 | 0 | 0 |
| A3_2 | Colwelliaceae | 0 | 0 | 0 | 0 | 0 | 0.31 ± 0.11 | 0 |
| A3_2 | Coriobacteriaceae | 0 | 0.6 ± 0.06 | 0 | 0 | 0 | 0 | 0 |
| A3_2 | Corynebacterineae | 0 | 0 | 0 | 0 | 0 | 7.65 ± 0.04 | 0 |
| A3_2 | Crocinitomicaceae | 0 | 0 | 0 | 0 | 2.7 ± 0.49 | 0 | 0 |
| A3_2 | Enterobacteriaceae | 1.17 ± 0.02 | 18.72 ± 0.51 | 0 | 1.94 ± 0.09 | 0.98 ± 0.36 | 0 | 0 |
| A3_2 | Erythrobacteraceae | 0 | 0 | 0 | 0 | 0 | 0 | 3.52 ± 1.37 |
| A3_2 | FCB cluster | 8.03 ± 0.38 | 4.25 ± 0.37 | 2.88 ± 0.06 | 2.96 ± 0.17 | 1.09 ± 0.21 | 1.1 ± 0.16 | 8.87 ± 0.54 |
| A3_2 | Flavobacteriaceae | 12.78 ± 0.69 | 10.79 ± 1.65 | 2.04 ± 0.01 | 0 | 13.97 ± 0.28 | 2.33 ± 0.17 | 15.95 ± 1.49 |
| A3_2 | Halieaceae | 0 | 0 | 0 | 0 | 9.83 ± 0.15 | 0 | 0 |
| A3_2 | Moraxellaceae | 0 | 0 | 0 | 0 | 0 | 9.53 ± 0.34 | 0 |
| A3_2 | Nitrosomonadaceae | 0.18 ± 0.05 | 0 | 0 | 3.07 ± 0.16 | 0 | 0 | 0 |
| A3_2 | Oceanospirillaceae | 0.98 ± 0.11 | 0 | 0 | 0 | 1.32 ± 0.11 | 0.76 ± 0.28 | 0.47 ± 0.01 |
| A3_2 | Other | 10.13 ± 0.23 | 14.12 ± 0.1 | 10.13 ± 0.42 | 16.24 ± 0.13 | 5.83 ± 0.31 | 9.44 ± 0.4 | 8.04 ± 0.67 |
| A3_2 | other Actinobacteria | 11.05 ± 0.26 | 3.1 ± 0.17 | 9.5 ± 0.08 | 3.34 ± 0.02 | 1.94 ± 0.09 | 8.55 ± 0.02 | 0.88 ± 0.06 |
| A3_2 | other Alphaproteobacteria | 10.4 ± 0.3 | 6.25 ± 0.15 | 5.55 ± 0.64 | 7.46 ± 0.05 | 5.59 ± 0.52 | 11.04 ± 0.18 | 7 ± 0.25 |
| A3_2 | other Betaproteobacteria | 5.22 ± 0.2 | 2.21 ± 0.43 | 4.78 ± 0.25 | 4.92 ± 0.07 | 6.88 ± 0.45 | 6.34 ± 0.34 | 1.56 ± 0.08 |
| A3_2 | other Cyanobacteria | 0.74 ± 0.06 | 0.78 ± 0.13 | 3.44 ± 0.04 | 1.15 ± 0.05 | 2.12 ± 0.64 | 0.09 ± 0.05 | 0.94 ± 0.01 |
| A3_2 | other Gammaproteobacteria | 17.96 ± 0.23 | 14.19 ± 0.91 | 11.62 ± 0.49 | 16.05 ± 0.29 | 14.49 ± 1.61 | 14.59 ± 0.4 | 12.65 ± 0.14 |
| A3_2 | other Proteobacteria | 3.36 ± 0.22 | 3.21 ± 0.04 | 0.3 ± 0.06 | 6.04 ± 0.04 | 3.35 ± 0.26 | 2.24 ± 0.14 | 1.41 ± 0.01 |
| A3_2 | Oxalobacteraceae | 1.03 ± 0.18 | 0 | 0 | 0 | 0 | 0 | 0 |
| A3_2 | Pelagibacteraceae | 5.84 ± 0.44 | 0 | 0 | 0 | 0 | 6.37 ± 0.15 | 0 |
| A3_2 | Phyllobacteriaceae | 0 | 0 | 3.16 ± 0.15 | 0 | 0 | 0 | 0 |
| A3_2 | Piscirickettsiaceae | 0 | 0 | 2.81 ± 0.03 | 0 | 0 | 0 | 0 |
| A3_2 | Prochloraceae | 0 | 0 | 3.05 ± 0.1 | 1.46 ± 0.35 | 0 | 0 | 0 |
| A3_2 | Pseudoalteromonadaceae | 2.04 ± 0.19 | 1.18 ± 0.03 | 0 | 0 | 0 | 6.88 ± 0.09 | 1.89 ± 0.06 |
| A3_2 | Pseudomonadaceae | 0 | 0 | 5.74 ± 0.03 | 2.66 ± 0.07 | 3.71 ± 0.41 | 0 | 0 |
| A3_2 | Rhizobiaceae | 0 | 0 | 2.43 ± 0.13 | 0 | 0 | 0 | 0 |
| A3_2 | Rhodobacteraceae | 0 | 8.34 ± 0.23 | 15.37 ± 0.4 | 0 | 2.84 ± 0.11 | 0 | 0 |
| A3_2 | SAR86 | 3.58 ± 0.02 | 0 | 0 | 0 | 0 | 0 | 0 |
| A3_2 | Shewanellaceae | 0 | 5.92 ± 0.33 | 2.22 ± 0.03 | 8.08 ± 0.12 | 1.71 ± 0.32 | 0 | 10.97 ± 0.41 |
| A3_2 | Sphingomonadaceae | 0 | 0 | 0 | 0 | 0 | 0 | 2.57 ± 0.1 |
| A3_2 | Synechococcaceae | 0 | 1.36 ± 0.06 | 1.5 ± 0.15 | 5.48 ± 0.18 | 9.16 ± 0.13 | 0 | 0 |
| A3_2 | Yersiniaceae | 0 | 2.65 ± 0.08 | 0 | 0 | 0 | 0 | 0 |
| FL | Actinomycetaceae | 0 | 0 | 1.93 ± 0.08 | 0 | 0 | 0 | 0 |
| FL | Aeromonadaceae | 0 | 0 | 0 | 4.67 ± 0.47 | 0 | 0 | 0 |
| FL | Alcanivoracaceae | 0 | 0 | 0 | 7.68 ± 0.55 | 0 | 0 | 0 |
| FL | Alteromonadaceae | 2.76 ± 0.22 | 0 | 0 | 2.65 ± 0.33 | 8.74 ± 0.67 | 6.79 ± 0.09 | 12.41 ± 0.31 |
| FL | Aphanizomenonaceae | 0 | 0 | 0 | 2.57 ± 0.53 | 0 | 0 | 0 |
| FL | Archaea | 0 | 2.86 ± 0.44 | 6.01 ± 0.28 | 1.74 ± 0.19 | 1.26 ± 0.08 | 0 | 0.44 ± 0.03 |
| FL | Bacillaceae | 0 | 0 | 2.93 ± 0.25 | 0 | 0 | 0 | 0 |
| FL | Burkholderiaceae | 2.37 ± 0.25 | 0 | 0 | 0 | 0 | 4.31 ± 0.09 | 1.5 ± 0.05 |
| FL | Cellvibrionaceae | 0 | 0 | 0 | 3.17 ± 0.18 | 4.2 ± 0.12 | 0 | 5.18 ± 0.11 |
| FL | Chromatiaceae | 0 | 0 | 1.64 ± 0.29 | 0 | 0 | 0 | 0 |
| FL | Chromobacteriaceae | 0 | 0 | 0 | 0 | 1.9 ± 0.35 | 0 | 0 |
| FL | Colwelliaceae | 0 | 0 | 0 | 0 | 0 | 5.92 ± 0.66 | 0 |
| FL | Coriobacteriaceae | 0 | 0.62 ± 0.21 | 0 | 0 | 0 | 0 | 0 |
| FL | Corynebacterineae | 0 | 0 | 0 | 0 | 0 | 2.3 ± 0.17 | 0 |
| FL | Crocinitomicaceae | 0 | 0 | 0 | 0 | 4.81 ± 0.97 | 0 | 0 |
| FL | Enterobacteriaceae | 4.61 ± 0.37 | 14.67 ± 0.18 | 0 | 1.3 ± 0.01 | 0.83 ± 0.04 | 0 | 0 |
| FL | Erythrobacteraceae | 0 | 0 | 0 | 0 | 0 | 0 | 3.94 ± 0.97 |
| FL | FCB cluster | 4.22 ± 0.5 | 4.95 ± 0.12 | 3.09 ± 0.11 | 2.43 ± 0.3 | 0.66 ± 0.03 | 0.36 ± 0.03 | 12.6 ± 0.63 |
| FL | Flavobacteriaceae | 7.38 ± 0.34 | 8.84 ± 0.38 | 2.14 ± 0.14 | 0 | 11.44 ± 0.65 | 0.72 ± 0.05 | 13.55 ± 0.94 |
| FL | Halieaceae | 0 | 0 | 0 | 0 | 9.28 ± 0.86 | 0 | 0 |
| FL | Moraxellaceae | 0 | 0 | 0 | 0 | 0 | 7.57 ± 0.92 | 0 |
| FL | Nitrosomonadaceae | 13.99 ± 1.42 | 0 | 0 | 3.96 ± 0.83 | 0 | 0 | 0 |
| FL | Oceanospirillaceae | 3.55 ± 0.34 | 0 | 0 | 0 | 4.36 ± 0.35 | 11.47 ± 0.47 | 0.82 ± 0.04 |
| FL | Other | 6.72 ± 0.07 | 13.71 ± 0.57 | 9.53 ± 0.17 | 12.93 ± 0.06 | 5.15 ± 0.93 | 7.73 ± 0.11 | 7.31 ± 0.17 |
| FL | other Actinobacteria | 7.46 ± 0.12 | 3.8 ± 0.04 | 8.72 ± 0.02 | 2.74 ± 0.38 | 0.9 ± 0.11 | 2.97 ± 0.1 | 1.22 ± 0.02 |
| FL | other Alphaproteobacteria | 8.14 ± 0.97 | 6.09 ± 0.24 | 5.81 ± 0.21 | 3.58 ± 0.5 | 4.58 ± 0.3 | 7.34 ± 0.04 | 7.23 ± 0.01 |
| FL | other Betaproteobacteria | 3.26 ± 0.05 | 5.77 ± 0.08 | 4.96 ± 0.12 | 6.24 ± 0.39 | 4.31 ± 0.25 | 8.05 ± 1.3 | 1.83 ± 0.07 |
| FL | other Cyanobacteria | 0.5 ± 0.02 | 1.06 ± 0.18 | 2.9 ± 0.18 | 1.41 ± 0.21 | 4.04 ± 0.38 | 0.03 ± 0.01 | 1.38 ± 0.06 |
| FL | other Gammaproteobacteria | 12.76 ± 0.38 | 15.74 ± 0.05 | 11.07 ± 0.04 | 13.12 ± 0.22 | 9.97 ± 0.9 | 25.6 ± 1.03 | 13.57 ± 0.21 |
| FL | other Proteobacteria | 1.79 ± 0.13 | 2.84 ± 0.36 | 0.25 ± 0.02 | 9.19 ± 0.51 | 1.36 ± 0.33 | 0.86 ± 0.03 | 1.56 ± 0.01 |
| FL | Oxalobacteraceae | 12.22 ± 1.55 | 0 | 0 | 0 | 0 | 0 | 0 |
| FL | Pelagibacteraceae | 4.09 ± 0.22 | 0 | 0 | 0 | 0 | 2.56 ± 0.03 | 0 |
| FL | Phyllobacteriaceae | 0 | 0 | 3.78 ± 0.05 | 0 | 0 | 0 | 0 |
| FL | Piscirickettsiaceae | 0 | 0 | 2.27 ± 0.08 | 0 | 0 | 0 | 0 |
| FL | Prochloraceae | 0 | 0 | 3.06 ± 0.01 | 1.58 ± 0.11 | 0 | 0 | 0 |
| FL | Pseudoalteromonadaceae | 1.84 ± 0.1 | 1.02 ± 0.06 | 0 | 0 | 0 | 5.5 ± 0.11 | 1.4 ± 0.02 |
| FL | Pseudomonadaceae | 0 | 0 | 4.6 ± 0.16 | 4.15 ± 0.35 | 7.88 ± 0.68 | 0 | 0 |
| FL | Rhizobiaceae | 0 | 0 | 2.37 ± 0.02 | 0 | 0 | 0 | 0 |
| FL | Rhodobacteraceae | 0 | 11.47 ± 1.07 | 18.96 ± 0.76 | 0 | 5.14 ± 0.27 | 0 | 0 |
| FL | SAR86 | 2.43 ± 0.08 | 0 | 0 | 0 | 0 | 0 | 0 |
| FL | Shewanellaceae | 0 | 2.91 ± 0.36 | 2.34 ± 0.13 | 9.47 ± 0.4 | 4.02 ± 0.33 | 0 | 10.81 ± 0.32 |
| FL | Sphingomonadaceae | 0 | 0 | 0 | 0 | 0 | 0 | 3.34 ± 0.05 |
| FL | Synechococcaceae | 0 | 1.32 ± 0.13 | 1.75 ± 0.03 | 5.52 ± 0.68 | 5.26 ± 0.6 | 0 | 0 |
| FL | Yersiniaceae | 0 | 2.42 ± 0.02 | 0 | 0 | 0 | 0 | 0 |
| R2 | Actinomycetaceae | 0 | 0 | 1.88 ± 0.1 | 0 | 0 | 0 | 0 |
| R2 | Aeromonadaceae | 0 | 0 | 0 | 2.37 ± 0.08 | 0 | 0 | 0 |
| R2 | Alcanivoracaceae | 0 | 0 | 0 | 3.91 ± 0.57 | 0 | 0 | 0 |
| R2 | Alteromonadaceae | 2.36 ± 0.07 | 0 | 0 | 1.13 ± 0.34 | 3.51 ± 0.61 | 3.09 ± 0.6 | 10.23 ± 0.87 |
| R2 | Aphanizomenonaceae | 0 | 0 | 0 | 13.61 ± 0.15 | 0 | 0 | 0 |
| R2 | Archaea | 0 | 3.46 ± 0.13 | 5.64 ± 0.12 | 1.02 ± 0.21 | 4.35 ± 0.79 | 0 | 0.64 ± 0.04 |
| R2 | Bacillaceae | 0 | 0 | 2.23 ± 0.18 | 0 | 0 | 0 | 0 |
| R2 | Burkholderiaceae | 3.74 ± 0.18 | 0 | 0 | 0 | 0 | 39.68 ± 6.11 | 1.41 ± 0.05 |
| R2 | Cellvibrionaceae | 0 | 0 | 0 | 2.06 ± 0.4 | 13.02 ± 12.37 | 0 | 5.45 ± 0.12 |
| R2 | Chromatiaceae | 0 | 0 | 3.5 ± 0.1 | 0 | 0 | 0 | 0 |
| R2 | Chromobacteriaceae | 0 | 0 | 0 | 0 | 1.74 ± 0.09 | 0 | 0 |
| R2 | Colwelliaceae | 0 | 0 | 0 | 0 | 0 | 0.07 ± 0.03 | 0 |
| R2 | Coriobacteriaceae | 0 | 3.15 ± 0.61 | 0 | 0 | 0 | 0 | 0 |
| R2 | Corynebacterineae | 0 | 0 | 0 | 0 | 0 | 3.39 ± 0.23 | 0 |
| R2 | Crocinitomicaceae | 0 | 0 | 0 | 0 | 4.52 ± 1.45 | 0 | 0 |
| R2 | Enterobacteriaceae | 1.1 ± 0.09 | 5.35 ± 0.04 | 0 | 4.04 ± 0.19 | 7.33 ± 1.12 | 0 | 0 |
| R2 | Erythrobacteraceae | 0 | 0 | 0 | 0 | 0 | 0 | 1.68 ± 0.36 |
| R2 | FCB cluster | 5.96 ± 0.32 | 3.93 ± 0.12 | 2.84 ± 0.13 | 2.21 ± 0.34 | 1.41 ± 0.49 | 0.37 ± 0.15 | 12.57 ± 0.13 |
| R2 | Flavobacteriaceae | 9.61 ± 2.28 | 6.56 ± 1.09 | 3.48 ± 0.17 | 0 | 15.91 ± 2.37 | 1.15 ± 0.2 | 12.24 ± 0.44 |
| R2 | Halieaceae | 0 | 0 | 0 | 0 | 2.16 ± 0.8 | 0 | 0 |
| R2 | Moraxellaceae | 0 | 0 | 0 | 0 | 0 | 0.88 ± 0.03 | 0 |
| R2 | Nitrosomonadaceae | 0.82 ± 0.11 | 0 | 0 | 1.57 ± 0.07 | 0 | 0 | 0 |
| R2 | Oceanospirillaceae | 0.88 ± 0.04 | 0 | 0 | 0 | 0.7 ± 0.19 | 0.23 ± 0.03 | 2.02 ± 0.15 |
| R2 | Other | 14.91 ± 0.03 | 12.59 ± 0.41 | 11.06 ± 0.04 | 6.32 ± 1.04 | 5.59 ± 0.07 | 5.31 ± 0.27 | 7.46 ± 0.36 |
| R2 | other Actinobacteria | 12.99 ± 0.01 | 3.08 ± 0.11 | 8.28 ± 0.07 | 1.43 ± 0.05 | 5.26 ± 0.67 | 6.65 ± 2.05 | 1.39 ± 0.14 |
| R2 | other Alphaproteobacteria | 7.71 ± 0.6 | 4.79 ± 0.25 | 7.49 ± 0.13 | 1.11 ± 0.06 | 7.43 ± 1.64 | 6.82 ± 0.88 | 8.52 ± 0.15 |
| R2 | other Betaproteobacteria | 6.44 ± 0.61 | 3.04 ± 0.03 | 4.65 ± 0.22 | 5.26 ± 0.13 | 4.72 ± 1.51 | 4.26 ± 0.15 | 1.86 ± 0.11 |
| R2 | other Cyanobacteria | 0.98 ± 0.08 | 1.42 ± 0.28 | 2.56 ± 0.14 | 6.37 ± 0.42 | 0.92 ± 0.01 | 0.08 ± 0.01 | 1.76 ± 0.47 |
| R2 | other Gammaproteobacteria | 14.69 ± 0.04 | 13.04 ± 0.44 | 9.96 ± 0.26 | 10.35 ± 1.09 | 7.8 ± 0.99 | 13.69 ± 1.85 | 15.29 ± 0.06 |
| R2 | other Proteobacteria | 3.66 ± 0.15 | 2.67 ± 0.06 | 0.48 ± 0.01 | 4.44 ± 0.48 | 1.36 ± 0.63 | 1.85 ± 0.32 | 1.66 ± 0.07 |
| R2 | Oxalobacteraceae | 1.2 ± 0.13 | 0 | 0 | 0 | 0 | 0 | 0 |
| R2 | Pelagibacteraceae | 9.63 ± 0.35 | 0 | 0 | 0 | 0 | 9.11 ± 0.07 | 0 |
| R2 | Phyllobacteriaceae | 0 | 0 | 6.69 ± 0.22 | 0 | 0 | 0 | 0 |
| R2 | Piscirickettsiaceae | 0 | 0 | 2.52 ± 0.25 | 0 | 0 | 0 | 0 |
| R2 | Prochloraceae | 0 | 0 | 4.18 ± 0.29 | 8.05 ± 0.39 | 0 | 0 | 0 |
| R2 | Pseudoalteromonadaceae | 0.86 ± 0.05 | 1.11 ± 0.08 | 0 | 0 | 0 | 3.44 ± 0.23 | 3.33 ± 0.15 |
| R2 | Pseudomonadaceae | 0 | 0 | 4.28 ± 0.14 | 0.82 ± 0.01 | 1.28 ± 0.41 | 0 | 0 |
| R2 | Rhizobiaceae | 0 | 0 | 2.44 ± 0.21 | 0 | 0 | 0 | 0 |
| R2 | Rhodobacteraceae | 0 | 9.18 ± 0.58 | 12.49 ± 0.33 | 0 | 1.88 ± 0.45 | 0 | 0 |
| R2 | SAR86 | 2.56 ± 0.46 | 0 | 0 | 0 | 0 | 0 | 0 |
| R2 | Shewanellaceae | 0 | 23.28 ± 1.11 | 1.24 ± 0.1 | 6.65 ± 0.24 | 0.65 ± 0.12 | 0 | 8.14 ± 0.54 |
| R2 | Sphingomonadaceae | 0 | 0 | 0 | 0 | 0 | 0 | 4.42 ± 0.12 |
| R2 | Synechococcaceae | 0 | 1.12 ± 0.07 | 2.21 ± 0.04 | 17.36 ± 1.73 | 8.56 ± 2.87 | 0 | 0 |
| R2 | Yersiniaceae | 0 | 2.31 ± 0.65 | 0 | 0 | 0 | 0 | 0 |
