# Supplementary material for: Microbial iron metabolism as revealed by gene expression profiles in contrasted Southern Ocean regimes
Source: Environ Microbiol. 2019 Apr 26;21(7):2360–74. doi: 10.1111/1462-2920.14621 (PMC6618146; doi:10.1111/1462-2920.14621)
Supplement: Supplementary file 11 — Supplementary Table 3. Taxa‐specific transcript abundance (per 105 cells) of a given pathway at Station F‐L and R‐2. For each group, mean value ± standard deviation of 2 replicates are shown. Error estimates are provided for all prokaryotic groups illustrated in Figs 6, and groups are listed by alphabetic order. [file EMI-21-2360-s011.docx]

**Supplementary Table 3.** Taxa-specific transcript abundance (per 10^5^ cells) of a given pathway at Station F-L and R-2. For each group, mean value ± standard deviation of 2 replicates are shown. Error estimates are provided for all prokaryotic groups illustrated in Figures 6, and groups are listed by alphabetic order.

| Station | Species | Pathway | Mean ± SD |
| --- | --- | --- | --- |
| F-L | Actinomycetales | Aconitase | 433.5 ± 55.87 |
| F-L | Actinomycetales | F2+ uptake | 39.81 ± 1.46 |
| F-L | Actinomycetales | F3+ uptake | 246.32 ± 14.14 |
| F-L | Actinomycetales | Flavodoxin switch | 9.4 ± 1.28 |
| F-L | Actinomycetales | Isocitrate lyase | 317 ± 7.08 |
| F-L | Actinomycetales | Ribosomal proteins | 24.1 ± 0.43 |
| F-L | Actinomycetales | Siderophore uptake | 2.09 ± 0.25 |
| F-L | Alteromonadales | Aconitase | 2804.42 ± 4.66 |
| F-L | Alteromonadales | F2+ uptake | 975.05 ± 29.47 |
| F-L | Alteromonadales | F3+ uptake | 1617.63 ± 24.8 |
| F-L | Alteromonadales | Flavodoxin switch | 961.79 ± 23.12 |
| F-L | Alteromonadales | Isocitrate lyase | 6742.55 ± 413.26 |
| F-L | Alteromonadales | Ribosomal proteins | 93235.65 ± 1342.4 |
| F-L | Alteromonadales | Bacterioferritin | 624.06 ± 8.33 |
| F-L | Alteromonadales | Siderophore uptake | 28433.39 ± 1163.07 |
| F-L | Burkholderiales | Aconitase | 146820 ± 21094.74 |
| F-L | Burkholderiales | F2+ uptake | 4002.1 ± 522.9 |
| F-L | Burkholderiales | F3+ uptake | 48495.46 ± 882.19 |
| F-L | Burkholderiales | Flavodoxin switch | 3505.73 ± 544.77 |
| F-L | Burkholderiales | Isocitrate lyase | 62830.09 ± 4936.64 |
| F-L | Burkholderiales | Ribosomal proteins | 314522.75 ± 48995.2 |
| F-L | Burkholderiales | Bacterioferritin | 2895.88 ± 107.54 |
| F-L | Burkholderiales | Siderophore uptake | 51850.49 ± 2905.22 |
| F-L | Flavobacteriales | Aconitase | 618 ± 2.83 |
| F-L | Flavobacteriales | F2+ uptake | 145.98 ± 7.65 |
| F-L | Flavobacteriales | F3+ uptake | 247.73 ± 21.77 |
| F-L | Flavobacteriales | Flavodoxin switch | 1.58 ± 0.59 |
| F-L | Flavobacteriales | Isocitrate lyase | 29.32 ± 1.95 |
| F-L | Flavobacteriales | Ribosomal proteins | 15925.98 ± 1438.44 |
| F-L | Flavobacteriales | Bacterioferritin | 85.51 ± 13.5 |
| F-L | Flavobacteriales | Siderophore uptake | 1809.17 ± 84.7 |
| F-L | Oceanospirillales | Aconitase | 1167.52 ± 0.07 |
| F-L | Oceanospirillales | F2+ uptake | 53.27 ± 1.51 |
| F-L | Oceanospirillales | F3+ uptake | 215.21 ± 12.69 |
| F-L | Oceanospirillales | Flavodoxin switch | 176.71 ± 6.33 |
| F-L | Oceanospirillales | Isocitrate lyase | 1239.78 ± 46.2 |
| F-L | Oceanospirillales | Ribosomal proteins | 40928.03 ± 290.1 |
| F-L | Oceanospirillales | Bacterioferritin | 101.06 ± 10.67 |
| F-L | Oceanospirillales | Siderophore uptake | 437.12 ± 2.45 |
| F-L | Pelagibacterales | Aconitase | 31.2 ± 0.43 |
| F-L | Pelagibacterales | F3+ uptake | 21.94 ± 1.08 |
| F-L | Pelagibacterales | Isocitrate lyase | 12.1 ± 0 |
| F-L | Pelagibacterales | Ribosomal proteins | 444.63 ± 4.14 |
| F-L | Pseudomonadales | Aconitase | 7598.56 ± 707.9 |
| F-L | Pseudomonadales | F2+ uptake | 2950.06 ± 291.64 |
| F-L | Pseudomonadales | F3+ uptake | 18795.22 ± 1129.27 |
| F-L | Pseudomonadales | Flavodoxin switch | 1675.58 ± 308.57 |
| F-L | Pseudomonadales | Isocitrate lyase | 40737.78 ± 1594.53 |
| F-L | Pseudomonadales | Ribosomal proteins | 441176.88 ± 3248.82 |
| F-L | Pseudomonadales | Bacterioferritin | 2417.7 ± 117.73 |
| F-L | Pseudomonadales | Siderophore uptake | 30927.55 ± 129.2 |
| F-L | Rhodobacterales | Aconitase | 155.47 ± 16.66 |
| F-L | Rhodobacterales | F2+ uptake | 80.35 ± 8 |
| F-L | Rhodobacterales | F3+ uptake | 461.17 ± 30.76 |
| F-L | Rhodobacterales | Flavodoxin switch | 8.9 ± 0.78 |
| F-L | Rhodobacterales | Isocitrate lyase | 40.91 ± 6.87 |
| F-L | Rhodobacterales | Ribosomal proteins | 4508.99 ± 712.13 |
| F-L | Rhodobacterales | Bacterioferritin | 21.15 ± 2.62 |
| F-L | Rhodobacterales | Siderophore uptake | 78.39 ± 0.19 |
| F-L | Synechococcales | Aconitase | 258.87 ± 17.67 |
| F-L | Synechococcales | F2+ uptake | 266.41 ± 32.84 |
| F-L | Synechococcales | F3+ uptake | 2748.01 ± 16.98 |
| F-L | Synechococcales | Flavodoxin switch | 563.17 ± 60.54 |
| F-L | Synechococcales | Ribosomal proteins | 105576.5 ± 6701.15 |
| F-L | Synechococcales | Bacterioferritin | 343.67 ± 30.96 |
| F-L | Synechococcales | Siderophore uptake | 1011.01 ± 30 |
| R-2 | Actinomycetales | Aconitase | 42193.15 ± 5722.64 |
| R-2 | Actinomycetales | F2+ uptake | 3194.07 ± 308.89 |
| R-2 | Actinomycetales | F3+ uptake | 18782.59 ± 1570.95 |
| R-2 | Actinomycetales | Flavodoxin switch | 617.87 ± 117.26 |
| R-2 | Actinomycetales | Isocitrate lyase | 35417.43 ± 2302.06 |
| R-2 | Actinomycetales | Ribosomal proteins | 5095.92 ± 161.52 |
| R-2 | Actinomycetales | Siderophore uptake | 272.33 ± 40.42 |
| R-2 | Alteromonadales | Aconitase | 71008.88 ± 10653.34 |
| R-2 | Alteromonadales | F2+ uptake | 176175.89 ± 7129.55 |
| R-2 | Alteromonadales | F3+ uptake | 52198.77 ± 161.71 |
| R-2 | Alteromonadales | Flavodoxin switch | 47663.54 ± 4736.17 |
| R-2 | Alteromonadales | Isocitrate lyase | 99877.15 ± 10599.23 |
| R-2 | Alteromonadales | Ribosomal proteins | 275256.83 ± 8632.97 |
| R-2 | Alteromonadales | Bacterioferritin | 7273.67 ± 136.36 |
| R-2 | Alteromonadales | Siderophore uptake | 1082336.94 ± 46623.43 |
| R-2 | Burkholderiales | Aconitase | 114020.04 ± 1920.06 |
| R-2 | Burkholderiales | F2+ uptake | 8350.3 ± 823.95 |
| R-2 | Burkholderiales | F3+ uptake | 105845.73 ± 1467.22 |
| R-2 | Burkholderiales | Flavodoxin switch | 11413.09 ± 1024.97 |
| R-2 | Burkholderiales | Isocitrate lyase | 427429.08 ± 66848.53 |
| R-2 | Burkholderiales | Ribosomal proteins | 138255.51 ± 9595.33 |
| R-2 | Burkholderiales | Bacterioferritin | 6040.08 ± 1212.87 |
| R-2 | Burkholderiales | Siderophore uptake | 99233.46 ± 1133.47 |
| R-2 | Flavobacteriales | Aconitase | 4657.41 ± 1319.02 |
| R-2 | Flavobacteriales | F2+ uptake | 1117.31 ± 279.88 |
| R-2 | Flavobacteriales | F3+ uptake | 2802.47 ± 173.62 |
| R-2 | Flavobacteriales | Flavodoxin switch | 53.45 ± 40.38 |
| R-2 | Flavobacteriales | Isocitrate lyase | 322.85 ± 47.01 |
| R-2 | Flavobacteriales | Ribosomal proteins | 58880.52 ± 1988.95 |
| R-2 | Flavobacteriales | Bacterioferritin | 862.24 ± 44.98 |
| R-2 | Flavobacteriales | Siderophore uptake | 13630.66 ± 752.65 |
| R-2 | Oceanospirillales | Aconitase | 4052.72 ± 426.93 |
| R-2 | Oceanospirillales | F2+ uptake | 390.74 ± 104.7 |
| R-2 | Oceanospirillales | F3+ uptake | 1541.95 ± 22.16 |
| R-2 | Oceanospirillales | Flavodoxin switch | 1414.24 ± 158.46 |
| R-2 | Oceanospirillales | Iso | 969.54 ± 221.75 |
| R-2 | Oceanospirillales | Ribosomal proteins | 21389.3 ± 671.28 |
| R-2 | Oceanospirillales | Bacterioferritin | 335.14 ± 53.06 |
| R-2 | Oceanospirillales | Siderophore uptake | 6786.09 ± 801.14 |
| R-2 | Pelagibacterales | Aconitase | 259.79 ± 18.48 |
| R-2 | Pelagibacterales | F3+ uptake | 537.07 ± 26.72 |
| R-2 | Pelagibacterales | Isocitrate lyase | 184.85 ± 0 |
| R-2 | Pelagibacterales | Ribosomal proteins | 4193.84 ± 261.32 |
| R-2 | Pseudomonadales | Aconitase | 1619.08 ± 34.74 |
| R-2 | Pseudomonadales | F2+ uptake | 645.59 ± 266.08 |
| R-2 | Pseudomonadales | F3+ uptake | 14064.86 ± 1143.46 |
| R-2 | Pseudomonadales | Flavodoxin switch | 771.49 ± 37.5 |
| R-2 | Pseudomonadales | Isocitrate lyase | 6623.61 ± 587.56 |
| R-2 | Pseudomonadales | Ribosomal proteins | 47072.01 ± 805.73 |
| R-2 | Pseudomonadales | Bacterioferritin | 354.52 ± 20.8 |
| R-2 | Pseudomonadales | Siderophore uptake | 25575.43 ± 921.16 |
| R-2 | Rhodobacterales | Aconitase | 5404.12 ± 67.84 |
| R-2 | Rhodobacterales | F2+ uptake | 5531.83 ± 155.66 |
| R-2 | Rhodobacterales | F3+ uptake | 22151.77 ± 1272.97 |
| R-2 | Rhodobacterales | Flavodoxin switch | 400.02 ± 17.43 |
| R-2 | Rhodobacterales | Isocitrate lyase | 708.99 ± 77.04 |
| R-2 | Rhodobacterales | Ribosomal proteins | 78689.53 ± 2421.74 |
| R-2 | Rhodobacterales | Bacterioferritin | 930.15 ± 100.77 |
| R-2 | Rhodobacterales | Siderophore uptake | 5932.9 ± 680.68 |
| R-2 | Synechococcales | Aconitase | 4082.61 ± 80.74 |
| R-2 | Synechococcales | F2+ uptake | 3334.5 ± 389.07 |
| R-2 | Synechococcales | F3+ uptake | 45383.65 ± 1140.23 |
| R-2 | Synechococcales | Flavodoxin switch | 59113.52 ± 3779.32 |
| R-2 | Synechococcales | Ribosomal proteins | 966188.35 ± 321.28 |
| R-2 | Synechococcales | Bacterioferritin | 4810.25 ± 484.25 |
| R-2 | Synechococcales | Siderophore uptake | 11052.27 ± 1219 |
