# Supplementary material for: Microbial iron metabolism as revealed by gene expression profiles in contrasted Southern Ocean regimes
Source: Environ Microbiol. 2019 Apr 26;21(7):2360–74. doi: 10.1111/1462-2920.14621 (PMC6618146; doi:10.1111/1462-2920.14621)
Supplement: Supplementary file 12 — Supplementary Table 4. Information of databases constructed or modified from Toulza et al. (2012) and retrieved by NCBI [file EMI-21-2360-s012.docx]

**Supplementary Table 4.** Information of databases constructed or modified from Toulza et al. (2012) and retrieved by NCBI

| Database Name | Specific processes referred to | Sum retrieved from NCBI/Moore Database | Global Ocean Sampling (GOS) protein database retrieval | Size of created and functionally verified (KEGG) databases in Fasta sequences | Mean length of sequences in customized database (bp) |
| --- | --- | --- | --- | --- | --- |
| Aconitase | TCA cycle | 527 | 807 | 1334 | 534 |
| Isocitrate lyase | Glyoxylate shunt | 1123 | 591 | 1714 | 412 |
| Fe | Fe^3+^ | 101 | 1150 | 1251 | 396 |
|  | Fe^2+^ | 243 | 757 | 1000 | 513 |
|  | Siderophore uptake | 561 | 2934 | 3495 | 513 |
|  | Flavodoxin switch | 181 | 886 | 1067 | 209 |
|  | Fe Storage | 123 | 427 | 550 | 170 |
