# Supplementary material for: Microbial iron metabolism as revealed by gene expression profiles in contrasted Southern Ocean regimes
Source: Environ Microbiol. 2019 Apr 26;21(7):2360–74. doi: 10.1111/1462-2920.14621 (PMC6618146; doi:10.1111/1462-2920.14621)
Supplement: Supplementary file 13 — Supplementary Table 5. Pathways and corresponding KEGG‐Id numbers that were chosen for further analysis [file EMI-21-2360-s013.docx]

| **Gene product** | **KEGG-ID** |
| --- | --- |
| Isocitrate lyase | K01637 |
| Aconitase | K01681, K01682 |
| Fe^3+^ | K02010, K02011, K02012, K02013, K11710 |
| Fe^2+^ | K07224, K07243, K11604, K11605, K11606, K11607, K11707, K16301, K04758, K04759 |
| Siderophore uptake | K02003, K02014, K02015, K02016, K02074, K02075, K02077, K03559, K03560, K03832, K06858, K16087, K16088, K16089 |
| Flavodoxin switch | K00230, K00380, K03809, K03839, K03840, K05524, K06205, K12264 |
| Fe Storage | K00522, K02217, K02255, K03594, K04047 |
| Ribosomal proteins | K003010 |
