# Supplementary material for: Microbial iron metabolism as revealed by gene expression profiles in contrasted Southern Ocean regimes
Source: Environ Microbiol. 2019 Apr 26;21(7):2360–74. doi: 10.1111/1462-2920.14621 (PMC6618146; doi:10.1111/1462-2920.14621)
Supplement: Supplementary file 14 — Supplementary Table 6. Cells per L for prokaryotic groups in Fig. 6 for station R‐2 and F‐L, calculated as described in experimental procedures. [file EMI-21-2360-s014.docx]

**Supplementary Table 6.** Cells per L for prokaryotic groups in Figure 6 for station R-2 and F-L, calculated as described in experimental procedures.

| Prokaryotic Order | R-2 | F-L |
| --- | --- | --- |
|  | cells L^−1^ | cells L^−1^ |
| Alteromonadales | 1045863.3 | 13983087.5 |
| Oceanospirillales | 11147372.2 | 27410029.5 |
| Pseudomonadales | 4336506.3 | 1032841.7 |
| Pelagibacterales | 102775200.0 | 165731367.0 |
| Flavobacteriales | 21478460.8 | 58156932.1 |
| Actinomycetales | 1096881.0 | 28125073.7 |
| Rhodobacterales | 5433387.3 | 119253491.0 |
| Burkholderiales | 162973.3 | 110346.3 |
| Synechococcales | 440264.9 | 1715609.3 |
